# Serum IL-15 in patients with early systemic sclerosis: a potential novel marker of lung disease

**DOI:** 10.1186/ar2284

**Published:** 2007-09-04

**Authors:** Dirk M Wuttge, Marie Wildt, Pierre Geborek, Frank A Wollheim, Agneta Scheja, Anita Åkesson

**Affiliations:** 1 Department of Rheumatology, Lund University Hospital, S-221 85 Lund, Sweden

## Abstract

The pathogenesis of systemic sclerosis (SSc) is characterized by autoimmunity, vasculopathy and fibrosis. IL-15 is a pleiotropic cytokine that has impact on immune, vascular and connective tissue cells. We therefore investigated IL-15 in the circulation of patients with early SSc and explored possible associations of serum IL-15 with vasculopathy and fibrosis. Serum levels of IL-15 were analysed in 63 consecutive patients with SSc of disease duration less than 4 years and without disease-modifying treatment. Thirty-three age-matched healthy control individuals were enrolled. Serum IL-15 levels were increased in the sera of SSc patients compared with that of healthy control individuals (*P *< 0.01). Serum IL-15 levels correlated with impaired lung function, assessed both by the vital capacity (*P *< 0.05) and by the carbon monoxide diffusion capacity (*P *< 0.05). The association between IL-15 and the vital capacity remained after multiple linear regression analysis. Patients with intermediate serum IL-15 levels had a higher prevalence of increased systolic pulmonary pressure compared with patients with either low or high serum IL-15 levels (*P *< 0.05). Moreover, increased serum IL-15 levels were associated with a reduced nailfold capillary density in multivariable logistic regression analysis (*P *< 0.01). Serum IL-15 levels also correlated inversely with the systolic blood pressure (*P *< 0.01). We conclude that IL-15 is associated with fibrotic as well as vascular lung disease and vasculopathy in early SSc. IL-15 may contribute to the pathogenesis of SSc. IL-15 could also be a candidate biomarker for pulmonary involvement and a target for therapy in SSc.

## Introduction

IL-15 is a cytokine of 14 to 15 kDa that belongs to the 4-a-helix bundle cytokine family, which also includes cytokines such as IL-2, IL-4 and IL-21. IL-15 signals through its specific IL-15 receptor α-chain that binds IL-15 with high affinity, and through the common IL-2 receptor γ-chain [1]. Recent studies have clarified that IL-15 is a survival and growth factor for T lymphocytes and B lymphocytes, for natural killer cells, for eosinophils and for mast cells [1-4]. IL-15 has emerged as an important molecule involved in autoimmunity and transplantation [5,6], and is considered a possible target for therapy in rheumatoid arthritis [7].

IL-15 mRNA is expressed in many tissues throughout the body, suggesting additional biologic functions outside the immune system. We have previously shown that IL-15 is expressed both by endothelial cells and by vascular smooth muscle cells in normal vessels [8]. IL-15 is also expressed in heart and skeletal muscle cells, in fibroblasts, in adipocytes, in epithelial cells as well as in keratinocytes [1,8]. IL-15 is expressed in the skin of TSK mice [9].

Several IL-15 signalling pathways are also implied in the putative pathogenesis of systemic sclerosis (SSc). IL-15 may contribute to autoimmunity via its effects on the activation and survival of T lymphocytes and B lymphocytes [2,3]. IL-15 may enhance perivascular infiltrates [10] and may induce CD44-mediated endothelial transmigration of lymphocytes [11]. IL-15 has also been shown to induce A1 and A2 arteriole contraction in a rat model [12]. A similar mechanism could lead to Raynaud's phenomena and other features of vasculopathy in SSc. In addition, IL-15 could contribute to the development of fibrosis by preventing apoptosis of collagen-producing myofibroblasts. Such a mechanism is supported by the observation that IL-15 can prevent TNFα-induced apoptosis of synovial fibroblasts in rheumatoid arthritis [13]. IL-15 has been shown to aggravate graft versus host disease [6], a disease with skin changes similar to SSc [14].

For all these reasons we considered it of interest to explore the occurrence of IL-15 in the circulation of patients with early SSc [15], with special regard to the possible association of IL-15 with vasculopathy and fibrosis.

## Materials and methods

### Patients and control individuals

From 1 January 1990 to 31 June 2005 serum samples were collected from consecutive patients, when they first presented to our unit, who fulfilled the inclusion criteria of: a definitive diagnosis of SSc according to the American College of Rheumatology [16]; a disease duration less than 4 years from the onset of skin involvement; and no previous treatment with any of the following drugs: azathioprine, chlorambucil, colchicine, cyclophosphamide, cyclosporine, D-penicillamine, methotrexate or mycophenolate mofetil.

Sixty-three patients (51 women, 12 men) with a median age of 54 years (range 23 to 78 years) and a median disease duration of 18 months (range 3 to 42 months) met these criteria. Forty-five patients (38 women, seven men) had limited cutaneous SSc, and 18 patients (13 women, five men) had proximal skin involvement and fulfilled the criteria for diffuse cutaneous SSc [17]. Fourteen patients were on treatment with a median (25th and 75th percentiles) of 10 mg (5 to 20 mg) prednisolone daily. Medications that may have influenced the systolic blood pressure at the time of evaluation consisted of calcium channel blockers (*n *= 26), angiotensin-converting enzyme inhibitors (*n *= 2), diuretics (*n *= 3), β-blockers (*n *= 2) or a combination thereof (*n *= 4). Smoking summarized all current smokers (*n *= 14) or previous smokers (*n *= 6), whereas nonsmokers included the remaining patients that had never smoked (*n *= 43). Thirty-three age-matched healthy control individuals were also enrolled. Informed consent was obtained from all participants.

### Clinical assessment

All clinical and laboratory data reported in this study were obtained within 1 week of blood sampling. The modified Rodnan skin score was evaluated by standardized palpation of the skin at 17 locations on the body and grading the skin thickness on a scale from 0 to 3, resulting in a maximum skin score of 51 points [18]. Nailfold capillaries were analysed quantitatively by direct microscopy counts of capillaries per millimetre on at least two fingers [19]. A capillary density below the second standard deviation of a normal population of 80 healthy individuals (<5.8 loops/mm) was regarded as pathologically reduced (A. Scheja, personal communication).

Oesophageal involvement was defined as distal hypomotility on cine radiography. Radiological lung involvement was defined as basal interstitial fibrosis on a plain chest X-ray scan or on high-resolution computer tomography. Lung function tests included assessment of the vital capacity (VC) by dry spirometry and of the diffusing capacity for carbon monoxide (DLCO) by the single-breath test. All values are depicted as the population percentage (p%). DLCO values less than 75 p% were regarded as reduced.

Cardiac involvement was assessed by radiological examination of the chest, by 12-lead electrocardiography and by Doppler cardiography, and was defined as pericarditis, an abnormal electrocardiography or cardiomegaly. Systolic pulmonary artery pressures of 40 mmHg and above determined by Doppler cardiography were regarded as pathologically increased.

Muscle involvement was defined as proximal muscle weakness or serum creatinine kinase levels elevated three times or more above the upper normal limit (3.3 μkat/l). Joint involvement was defined as palpable synovitis.

Renal involvement was defined as a decreased glomerular filtration rate <70 p%, either assessed by ^51^Cr-ethylenediamine tetraacetic acid and iohexol clearance [20] or calculated from serum cystatin C levels [21].

Antinuclear antibodies were analysed by an indirect immunofluorescence technique using the human Hep-2 cell line as the substrate [22]. Inflammatory activity was defined as either an increased erythrocyte sedimentation rate (≥15 and ≥22 mm/hour for men below and above age 50 years, respectively; ≥24 and ≥32 mm/hour for women below and above age 50 years, respectively), an increased C-reactive protein level (≥3 mg/l), or an increased orosomucoid level (>1.17 g/l).

### Measurement of IL-15

IL-15 was assayed with a commercial human IL-15 ELISA (R&D Systems, Minneapolis, MN, USA), following the manufacturer's instructions. Each serum sample was tested in duplicate. A linear detection range of the ELISA above 0.1 pg/ml has previously been reported [23]. The detection threshold was set above the blank value.

### Statistical analyses

Data were analysed with STATISTICA version 6 (StatSoft, Tulsa, OK, USA) and are depicted as the median (25th and 75th percentiles). The Kruskal–Wallis test was used for multiple group comparison, before the Mann–Whitney test was used for comparison between two groups. The chi-square test for 2 × 3 tables was used when applicable. Frequencies between groups were calculated by Fisher's exact test. All variables and groups as well as residuals in multiple linear regression analyses were analysed for a normal distribution and were tested by the Shapiro–Wilk test. W values >0.93 were accepted as normally distributed. Spearman's (*r*_s_) and partial correlations as well as multiple linear regression analyses were used to estimate correlations.

Data are expressed as the partial correlation coefficient (*r*), the regression coefficient (β_est_), the 95% confidence interval and as the coefficient of determination. For continuous variables, the standardized β_est _value and the 95% confidence interval is depicted. Results of multivariable logistic regression are depicted as the odds ratio and the 95% confidence interval. Probability *P *values (two-sided) were considered significant when <0.05.

## Results

### Serum IL-15 levels

**Figure 1:**
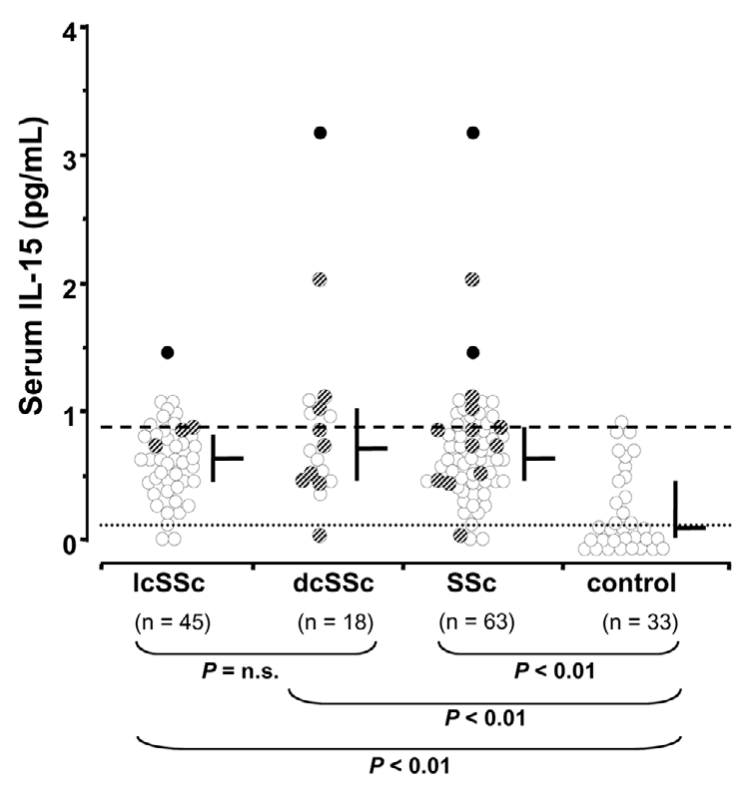
Serum concentrations of IL-15 in patients with systemic sclerosis increase compared with healthy control individuals. Striped circles, patients on cortisone treatment; filled circles, two patients with renal crises; horizontal bars, median (25th and 75th percentiles) in each group; dashed line, cut-off value for increased serum levels of IL-15 (75th percentile of all systemic sclerosis (SSc)); fine dotted line, detection level. dcSSc, diffuse cutaneous systemic sclerosis; lcSSc, limited cutaneous systemic sclerosis; n.s., not significant.

Increased serum IL-15 levels were observed in SSc patients (0.63 (0.47 to 0.88) pg/ml) compared with the healthy individuals (0 (0 to 0.46) pg/ml) (Figure 1). This applied to IL-15 levels in patients with limited cutaneous SSc (0.63 (0.45 to 0.83) pg/ml) and diffuse cutaneous SSc (0.72 (0.47 to 1.03) pg/ml) compared with control individuals, but the IL-15 levels did not differ between limited cutaneous SSc and diffuse cutaneous SSc.

Three outliers were identified in the SSc patient group. Two of these outliers were excluded from further analysis since both were derived from patients in renal crisis, one of which was already undergoing haemodialysis, and the reduced renal function might impede IL-15 elimination. The third outlier, however, had no identifiable confounding factors and was therefore included in the analysis.

Serum IL-15 levels of the 61 studied patients fulfilled the criteria for a normal distribution. Fourteen out of 33 control individuals had detectable levels of IL-15. These individuals did not take any medications; neither did they have any accompanying disease or did they differ in age from the other control individuals.

### Low, intermediate and high serum IL-15 levels describe three subsets of SSc patients

Low, intermediate and high serum IL-15 levels depict three subsets of SSc patients regarding the skin score, the VC, the DLCO and the systolic pulmonary arterial pressure. Table 1 presents the findings of patients with low, intermediate and high levels of IL-15.

**Table 1:** Characteristics of systemic sclerosis patients with low, intermediate and high serum IL-15 levels

Characteristic	Low IL-15 level, <0.64 pg/ml (*n *= 33)		Intermediate IL-15 level, 0.64–0.88 pg/ml (*n *= 15)		High IL-15 level, >0.88 pg/ml (*n *= 13)	
Female/male	29/4	(88/12)	11/4	(73/27)	10/3	(77/23)
Age at onset (years)	52	(45–57)	48	(45–54)	57	(53–61)
Disease duration (months)	19	(13–28)	16	(11–25)	11	(8–21)
Clinical features						
Skin score (points)	9	(4–14)	11	(4–17)	15	(8–21)
Diffuse cutaneous systemic sclerosis/limited cutaneous systemic sclerosis	8/25	(24/76)	3/12	(20/80)	6/7	(46/54)
Pitting scars or ulcer	9	(27)	6	(40)	3	(23)
Telangiectasis	9	(27)	6	(40)	1	(8)
Calcinosis	6	(18)	5	(33)	5	(38)
Organ involvement						
Oesophagus	20	(61)	11	(73)	10	(77)
Lung (radiological)	7	(21)	2	(13)	4	(31)
Heart	5	(15)	4	(27)	4	(31)
Kidney	1	(3)	1	(7)	0	(0)
Muscle	1	(3)	2	(13)	1	(8)
Joint	5	(15)	1	(7)	0	(0)
Lung function tests						
Vital capacity (p%)	94	(85–103)	94	(84–105)	75	(66–89)*
Diffusing capacity for carbon monoxide (p%)	84	(77–97)	71	(60–87)^†^	70	(62–81)^‡^
Serological findings						
Antinuclear antibodies	21	(64)	14	(93)	11	(85)
Anti-scleroderma-70 antibodies	6	(18)	4	(27)	2	(15)
Anticentromer antibodies	10	(30)	2	(13)	2	(15)
Laboratory findings						
Erythrocyte sedimentation rate (mm/hour)	17	(10–27)	12	(8–22)	12	(6–18)
C-reactive protein (mg/l)	0	(0–6)	0	(0–8)	1.6	(0–8.1)
Orosomucoid (g/l)	0.8	(0.68–0.91)	0.86	(0.77–0.96)	0.85	(0.80–1.11)
IgG (g/l)	10.9	(9.5–14.6)	9.5	(7.6–12.2)	12.6	(9.6–15.1)

Data presented as the median (25th and 75th percentiles) or as *n *(%). Kruskal–Wallis analysis showed *P *< 0.05 for the vital capacity and diffusing capacity for carbon monoxide. *P < 0.05 for high IL-15 serum level versus low and intermediate IL-15 serum levels; ^‡^*P *< 0.05 for high IL-15 serum level versus low IL-15 serum level; ^†^*P *= 0.057 for intermediate IL-15 serum level versus low IL-15 serum level.

The 75th percentile of all SSc patients (0.88 pg/ml) was chosen as the cut-off value for high serum levels of IL-15 because serum IL-15 levels in the control individuals were not normally distributed in accordance with other studies [24] (Figure 1). In the group with high serum IL-15 levels, diffuse cutaneous SSc was twice as prevalent as limited cutaneous SSc. Among patients with low or intermediate serum IL-15 levels, limited cutaneous SSc predominated. The VC was significantly lower among patients with high IL-15 levels (*P *< 0.05).

**Figure 2:**
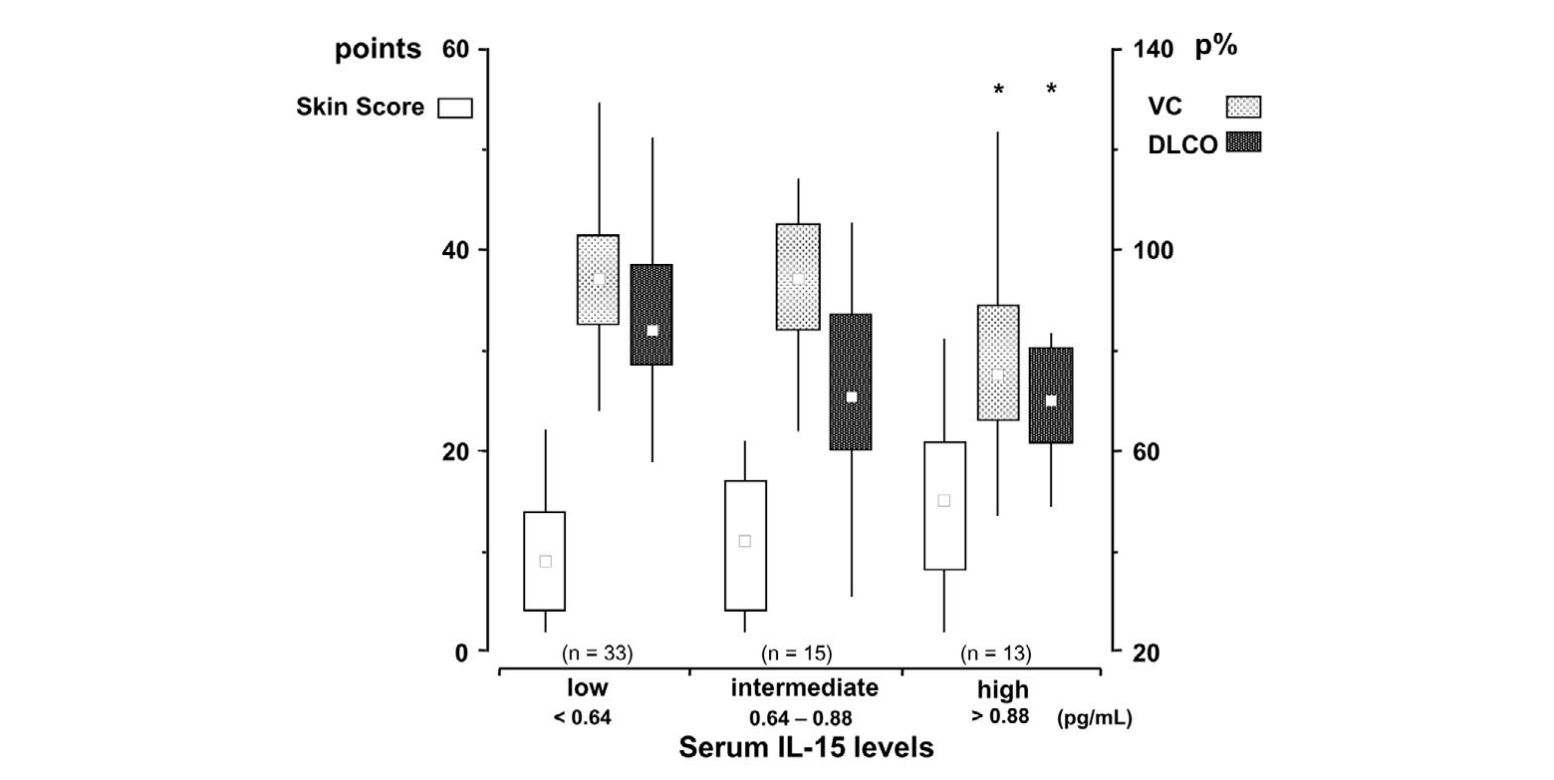
Low, intermediate and high serum IL-15 levels describe three groups of systemic sclerosis patients. Low, intermediate and high serum IL-15 levels depict three groups of systemic sclerosis patients with different skin score (white bars), different vital capacity (VC; grey bars) and different diffusing capacity for carbon monoxide (DLCO; black bars). Bars depict the median (25th and 75th percentile), and lines depict the 10th and 90th percentiles. Kruskal–Wallis analysis showed *P *< 0.05 for VC and DLCO with *P *= not significant for skin score. *P < 0.05 for VC of high IL-15 serum level versus low and intermediate IL-15 serum levels. *P < 0.05 for DLCO of high IL-15 serum level versus low IL-15 serum level. *P *= 0.057 for DLCO intermediate IL-15 serum level versus low IL-15 serum level. p%, population percentage.

We tested the possibility that a lower dividing point between low IL-15 levels and high IL-15 levels could change the characteristics of our patient groups. The median value of the SSc patients (0.63 pg/ml) was chosen as alternative cut-off level. Twenty-eight patients with serum IL-15 levels ≥0.64 pg/ml were compared with 33 patients with serum IL-15 levels <0.64 pg/ml. Lower DLCO levels (*P *< 0.01) and a higher prevalence of reduced finger capillary density (*P *< 0.05) were found in patients with serum IL-15 levels ≥0.64 pg/ml. The skin score, the SSc type or the VC showed no difference. Grouping the patients into those with low (<0.64 pg/ml), intermediate (0.64 to 0.88 pg/ml) and high (>0.88 mg/ml) serum IL-15 levels, however, showed that the group with high serum IL-15 levels had both low VC and low DLCO (Table 1). The intermediate group had a tendency to lower DLCO compared with the group with low serum IL-15 levels (Figure 2), although the two groups had similar skin scores or VC levels. The intermediated group also had a higher prevalence of patients with reduced DLCO (<75 p%) compared with the group with low serum IL-15 levels (53% versus 21%, *P *< 0.05). In addition, the VC/DLCO ratio tended to be higher in the intermediate serum level group, indicating predominating pulmonary vascular disease. Pulmonary hypertension (defined as systolic pulmonary pressure ≥40 mmHg) was found in four out of eleven patients (36%) in the intermediate IL-15 group, compared with one out of 31 patients (3%; *P *< 0.05) in the low IL-15 level group and zero out of 13 patients (0%; *P *< 0.05) in the high IL-15 group.

### Association between IL-15 and lung function

IL-15 correlated negatively with the VC (*r*_s _= -0.31, *P *< 0.05). This correlation became even more significant after statistical consideration of the presence of cortisone treatment by partial correlation (*r *= -0.42, *P *< 0.001). Multiple linear regression analysis of laboratory variables that may influence the VC showed that only IL-15 correlated inversely with the VC, whereas the presence of anticentromer antibodies was associated with high VC (Table 2). All associations remained significant after adjustment for multiple correlations and for smoking.

**Table 2:** Association of vital capacity with serum markers by multiple linear regression analysis

	β_est _(95% confidence interval)	*r*	*P*
IL-15	-0.36 (-0.57 to -0.14)	-0.40	0.002
Inflammatory activity	1.65 (-6.45 to 9.75)	0.05	0.691
Anticentromer antibodies	19.49 (9.72 to 29.27)	0.46	<0.001
Anti-scleroderma-70 antibodies	-0.60 (-10.49 to 9.30)	-0.02	0.605

Coefficient of determination = 37%. Inflammatory activity describes an increased erythrocyte sedimentation rate, increased C-reactive protein or increased orosomucoid either alone or in combination.

IL-15 also correlated inversely to the DLCO (*r*_s _= -0.33, *P *< 0.05), and the correlation remained after adjustment for cortisone (*r *= -0.32, *P *< 0.05). Applying multiple linear regression analysis of IL-15 and the DLCO including autoantibodies and inflammatory parameters, the inverse relation did not remain significant after adjustment for multiple correlations.

When the two patients with renal crisis were included in the analyses, however, the multiple linear regression analyses of IL-15 against both the VC and the DLCO became strong (*r *= -0.54, *P *< 0.001 for IL-15 versus VC; *r *= -0.46, *P *< 0.001 for IL-15 versus DLCO).

### Relation of IL-15 to nailfold capillary density

**Figure 3:**
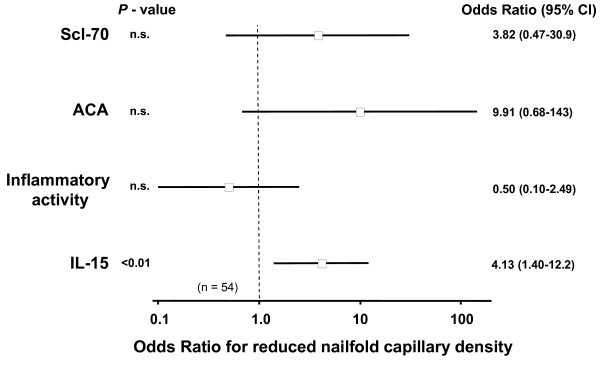
Relation between different serum markers and the occurrence of a reduced nailfold capillary density. Serum IL-15 correlated with an increased risk of reduced nailfold capillary density when compared with systemic-sclerosis-related antibodies and markers for inflammatory activity. Inflammatory activity describes an increased erythrocyte sedimentation rate, increased C-reactive protein or increased orosomucoid either alone or in combination. ACA, anticentromer antibody; 95% CI, 95% confidence interval; n.s., not significant; Scl-70, anti-scleroderma-70 antibody.

Forty-two out of 54 examined patients showed a reduced nailfold capillary density and had significantly increased serum IL-15 levels (0.72 (0.49 to 0.90) pg/ml) compared with the remaining 12 patients (0.47 (0.27 to 0.61) pg/ml) (*P *= 0.010). Serum IL-15 levels were identified as an important variable for the occurrence of reduced nailfold capillary density using multivariable logistic regression analysis (Figure 3). This correlation remained significant after adjustment for multiple correlations.

### Association between IL-15 and blood pressure

IL-15 correlated inversely with the systolic blood pressure by multiple linear regression analysis including variables that may influence the systolic blood pressure (Table 3). Age and the body mass index showed the expected positive correlation with systolic blood pressure. These data remained significant after adjustment for multiple correlations.

**Table 3:** Association of systolic blood pressure with several factors by multiple linear regression analysis

	β_est _(95% confidence interval)	*r*	*P*
Age	0.36 (0.15 to 0.58)	0.42	0.002
IL-15	-0.29 (-0.49 to -0.10)	-0.37	0.006
Blood pressure treatment	2.86 (-4.79 to 10.51)	0.10	0.467
Smoking	5.50 (-2.35 to 13.35)	0.19	0.176
Body mass index	0.36 (0.14 to 0.57)	0.41	0.002

Coefficient of determination = 48%.

## Discussion

Interstitial lung disease and pulmonary hypertension are currently the major causes of death in SSc [25]. Early diagnosis is crucial for initiation of treatment for SSc patients. In the present study, serum IL-15 levels were strongly negatively correlated with the VC. This liaison remained even after taking into account the presence of inflammatory markers or SSc-related antibodies that may influence the VC. This finding suggests a profibrotic activity of IL-15. In contrast, anticentromer antibodies correlated positively with the VC, which is in accordance with previous data showing that patients with anticentromer antibodies are relatively protected from pulmonary fibrosis [26]. Increased serum IL-15 levels have previously been found in sera of patients with various rheumatic diseases and interstitial pneumonia, including seven patients with SSc [23].

Fibrosis leading to restrictive interstitial lung disease and increased skin thickness is the hallmark of SSc. IL-15 may enhance perivascular inflammatory infiltrates by activation of endothelial cells to promote CD44-mediated extravasation of inflammatory cells [11]. Perivascular infiltrates are commonly seen early in SSc and are considered important for the initiation of the fibrotic response [10]. IL-15 has also been shown to prevent TNFα-mediated apoptosis of fibroblasts [13]. IL-15 may therefore promote myofibroblast survival and may increase production of the extracellular matrix.

Isolated reduction of the DLCO and an increased VC/DLCO ratio is highly suggestive for pulmonary hypertension primarily of vascular origin [26]. An intriguing observation was that the group of patients with intermediate serum IL-15 levels differed from the patients with low serum IL-15 levels by having a higher prevalence of reduced DLCO in concert with an unchanged skin score and VC as well as a higher VC/DLCO ratio, suggesting isolated pulmonary vascular involvement. The patients also had a high prevalence of increased systolic pulmonary pressure. Intermediate serum IL-15 levels therefore appeared to reflect pulmonary vascular involvement whereas high levels of serum IL-15 were associated with restrictive interstitial lung disease. Further studies are needed to confirm and explain the finding that only the group with an intermediate IL-15 serum level was associated with pulmonary hypertension whereas the group with high serum IL-15 levels did not show this relation.

Raynaud's phenomenon and the loss of capillaries are characteristic features of SSc [27]. In our cohort, patients with decreased nailfold capillary density had markedly increased serum IL-15 levels. Multiple logistic regression analysis showed that increased serum IL-15 levels were associated with a reduction in nailfold capillary density even after adjustment for inflammatory activity and the presence of SSc-associated antibodies. This indicated an independent influence of IL-15 on the vasculature. The direct impact of IL-15 on the microvasculature has been studied in rats [12]. Application of IL-15 locally or systemically resulted in strong contraction of A1 and A2 arterioles, which could not be reversed by addition of acetylcholine or nitroprusside. Our data suggest that IL-15 may participate in microvascular remodelling and a similar effect of IL-15 on A1 and A2 arterioles may possibly occur in our patients.

Serum IL-15 levels correlated negatively with the systolic blood pressure, even after adjustment for potential confounders such as age, body mass index, smoking or antihypertensive medication. One previous study evaluated the effects of IL-15 on cardiovascular complication in patients with hypertension [28]. In that study, IL-15 was increased in patients with severe organ damage even though the systolic blood pressure in these patients was slightly lower than in the group without organ damage. IL-15 was therefore identified as an independent risk factor for cardiovascular complication. This is particular of interest considering the accelerated atherosclerosis that has been found in SSc [29].

Importantly, IL-15 has previously been shown to reduce both blood pressure and the heart rate in a rat model when administered both locally and systemically, despite contraction of A1 and A2 arterioles [12]. This effect was attributed to reduced cardiac output. Accordingly, the inverse relation we observed between serum IL-15 and systolic blood pressure may indicate that IL-15 is active below picogramme levels in the circulating blood of patients. The blood pressure lowering effect might thus also be the result of reduced cardiac output and not the consequence of a reduced peripheral resistance. On the other hand, it is probable that the blood pressure lowering effect of IL-15 can be overridden by, for example, activation of the angiotensin–renin system, as illustrated by our two patients with renal crisis who had very high levels of IL-15 and concomitant high blood pressure.

Finally, serum IL-15 levels of our patients with early SSc correlated positively to serum creatinine kinase levels (*r*_s _= 0.32; *P *< 0.05). This indicates the possibility that IL-15 in serum may in part be derived from skeletal muscle and may reflect activity in terms of myopathy [30], since IL-15 is expressed by skeletal muscles [1]. Other probable sources, however, are alveolar macrophages [31], monocytes and fibroblasts in the skin – since IL-15 mRNA is upregulated in skin biopsies of the tight skin mouse [9] and in SSc-derived skin fibroblasts *in vitro *[32].

## Conclusion

Our data suggest that IL-15 may be a novel cytokine contributing to the pathogenesis of SSc, which would be in line with the capability of IL-15 to interact with several steps in the pathogenesis of SSc, such as vessel wall and fibroblast function. IL-15 may also be a potential biomarker for the disease. Studies are indicated to define the putative molecular mechanisms by which IL-15 may contribute to SSc. IL-15 should be considered a putative target for treatment of SSc, perhaps using an anti-human IL-15 antibody that is under development in a clinical phase II trial for the treatment of rheumatoid arthritis [7].

## Abbreviations

β_est_ = regression coefficient; DLCO = diffusing capacity for carbon monoxide; ELISA = enzyme-linked immunosorbent assay; IL = interleukin; p% = population percentage; *r* = partial correlation coefficient; *r*_s_ = Spearman’s correlation coefficient; SSc = systemic sclerosis; TNF = tumour necrosis factor; VC = vital capacity.

## Competing interests

The authors declare that they have no competing interests.

## Authors' contributions

DMW conceived the study, performed the statistical analysis, participated in its design, interpretation and coordination, and drafted the manuscript. MW carried out the immunoassay, and participated in the study design and in the revision of the manuscript. PG performed extraction of the patient data from the patient database, and participated in the statistical analysis and in the interpretation of the data. FAW participated in the study design, was involved in the revision of the manuscript and provided important intellectual content. AS participated in the design and coordination of the study, in the interpretation of the data and in the revision of manuscript. AA participated in the design and coordination of the study, in the interpretation of the data and in the revision the manuscript. All authors read and approved the final manuscript.
